# Quantitative trait locus linkage analysis in a large Amish pedigree identifies novel candidate loci for erythrocyte traits

**DOI:** 10.1002/mgg3.16

**Published:** 2013-05-31

**Authors:** Jesse D Hinckley, Diana Abbott, Trudy L Burns, Meadow Heiman, Amy D Shapiro, Kai Wang, Jorge Di Paola

**Affiliations:** 1 Department of Pediatrics and Human Medical Genetics and Genomics Program, University of Colorado Anschutz Medical CampusAurora, Colorado; 2 Duke Biostatistics Core, Duke UniversityDurham, North Carolina; 3 Departments of Epidemiology, College of Public Health, University of IowaIowa City, Iowa; 4 Departments of Indiana Hemophilia and Thrombosis CenterIndianapolis, Indiana; 5 Departments of Biostatistics, College of Public Health, University of IowaIowa City, Iowa

**Keywords:** Amish, erythrocytes, linkage, QTL

## Abstract

We characterized a large Amish pedigree and, in 384 pedigree members, analyzed the genetic variance components with covariate screen as well as genome-wide quantitative trait locus (QTL) linkage analysis of red blood cell count (RBC), hemoglobin (HB), hematocrit (HCT), mean corpuscular volume (MCV), mean corpuscular hemoglobin (MCH), mean corpuscular hemoglobin concentration (MCHC), red cell distribution width (RDW), platelet count (PLT), and white blood cell count (WBC) using SOLAR. Age and gender were found to be significant covariates in many CBC traits. We obtained significant heritability estimates for RBC, MCV, MCH, MCHC, RDW, PLT, and WBC. We report four candidate loci with Logarithm of the odds (LOD) scores above 2.0: 6q25 (MCH), 9q33 (WBC), 10p12 (RDW), and 20q13 (MCV). We also report eleven candidate loci with LOD scores between 1.5 and <2.0. Bivariate linkage analysis of MCV and MCH on chromosome 20 resulted in a higher maximum LOD score of 3.14. Linkage signals on chromosomes 4q28, 6p22, 6q25, and 20q13 are concomitant with previously reported QTL. All other linkage signals reported herein represent novel evidence of candidate QTL. Interestingly rs1800562, the most common causal variant of hereditary hemochromatosis in *HFE* (6p22) was associated with MCH and MCHC in this family. Linkage studies like the one presented here will allow investigators to focus the search for rare variants amidst the noise encountered in the large amounts of data generated by whole-genome sequencing.

## Introduction

Complete blood counts (CBC) are one of the most commonly ordered laboratory tests. Standard CBC analyses report several erythrocyte measures including red blood cell count (RBC), hemoglobin levels (HB), hematocrit (HCT), mean corpuscular volume (MCV), mean corpuscular hemoglobin (MCH), mean corpuscular hemoglobin concentration (MCHC) and red blood cell distribution width (RDW) as well as platelet count (PLT) and white blood cell count (WBC). In addition to providing a cursory overall evaluation of current status, several associations have been reported between CBC traits and thrombotic disease. RBC and HCT demonstrate association with increased risk of cardiovascular disease (CVD); HCT and HB are risk factors for ischemic heart disease; MCV is a risk factor for peripheral artery disease (PAD); and RDW is associated with increased risk of myocardial infarction, heart failure, and stroke (Haltmayer et al. 2002; Puddu et al. 2002; Felker et al. 2007; Tonelli et al. 2008; Ye et al. 2011; Ozsu et al. 2012). Furthermore, HCT is associated with morbidity and mortality in CVD and RDW is associated with higher mortality rates in cases of heart failure, pulmonary emboli, and PAD (Gagnon et al. 1994; Felker et al. 2007; Tonelli et al. 2008; Ye et al. 2011; Ozsu et al. 2012). Platelets play an important role in acute myocardial infarction (AMI) pathogenesis; furthermore, baseline PLT at the time of diagnosis is a strong predictor of reocclusion, reinfarction, and mortality even 1 year post-AMI (Nikolsky et al. 2007). WBC is an independent risk factor for the development of new coronary heart disease and a prognostic marker in existing CVD (Madjid et al. 2004).

CBC traits are multifactorial phenotypes determined by complex etiologies that display significant interperson variation as well as intraperson longitudinal stability (Costongs et al. 1985; Evans et al. 1999). Direct and inverse correlations have been well documented among erythrocyte traits including RBC, HB, HCT, and MCV (Evans et al. 1999). Intraindividual longitudinal stability and strong correlations among hematologic traits suggest these traits are subjected to homeostatic regulation, which is influenced by both environmental and genetic factors. There are also independent environmental modifiers of CBC traits including smoking, alcohol consumption, and use of oral contraceptives (Chalmers et al. 1979; Schwartz and Weiss 1991; Whitehead et al. 1995). Likewise, gender, age, and BMI are documented modifiers of multiple CBC traits including RBC, MCV, and WBC (Chalmers et al. 1979; Yokoyama and Akiyama 1995; Evans et al. 1999). Multiple variance components analyses report heritability estimates ranging from 0.10 to 0.86 for RBC, HB, HCT, MCV, MCH, MCHC, WBC, and PLT (Whitfield and Martin 1985; Dal Colletto et al. 1993; Garner et al. 2000). Though heritability estimates have varied, there is evidence for both a significant genetic component and a significant environmental component for all standard CBC traits (with the exception of RDW which has not been previously evaluated) (Evans et al. 1999).

Multiple genome-wide association and linkage studies have been published to date, reporting candidate quantitative trait loci (QTL) of CBC traits. Within Caucasian, African-American, and Japanese population cohorts, associations with different erythrocyte traits were reported on chromosomes 6p22 (*HFE*) and 16p13.3 (Ganesh et al. 2009; Kamatani et al. 2010; Lo et al. 2011). Additionally, the hemoglobin B gene cluster (chromosome 11p15.4) was identified as a candidate QTL of erythrocyte traits in Caucasian and African-American population cohorts (Lin et al. 2005; Yang et al. 2007; Lo et al. 2011). In multiple Caucasian and Japanese population cohorts, candidate QTL have been mapped for multiple erythrocyte traits to chromosomes 3q29 (*TFRC*), 6q23.3 (*HBS1L*, *MYB*), 6q24.1 (*CITED2*), 10p11.21, 12p24, and 22q12.3 (*TMPRSS6*) (Yang et al. 2007; Ganesh et al. 2009; Kamatani et al. 2010; Lo et al. 2011). For a comprehensive review of candidate QTL of CBC traits, see supporting information.

One approach to identifying QTL for complex traits like the CBC traits is to focus on a single large, culturally isolated pedigree where it is reasonable to assume that the genetic component represents the common variation of an outbred population while being under the control of fewer QTL (Heutink and Oostra 2002; Blangero et al. 2003). Thus, it is anticipated that related individuals with similar trait values will have a higher than expected amount of sharing of genetic material identical by descent (IBD) near the gene or genes that contribute to the quantitative trait. Additionally, genetic isolates demonstrate a more homogenous environmental component whereas a collection of ancestrally unrelated smaller pedigrees tend to have more variable life styles. We identified, characterized, and collected blood samples from a single Amish pedigree diagnosed with von Willebrand disease (VWD). CBC traits in this pedigree show variability that is representative of the normal population. QTL analysis of the pedigree identified several chromosomal regions that appear to harbor QTLs for CBC traits as reported herein. In addition to identifying novel candidate loci, we also report independent replication of three previously reported loci. This pedigree is a unique source of genetic information.

## Methods

### Amish pedigree and CBC

The Amish pedigree is composed of 821 individuals, of whom 395 provided blood samples. Seventy-four individuals are heterozygous for a C>T missense mutation at position 4120 (C4120T) in *VWF* that results in an arginine to cysteine amino acid substitution at position 1374 (R1374C) resulting in VWD type 2. The number of examined pedigree members included in the analysis presented herein is 384; eleven pedigree members were not included because of their uncertain position in the pedigree. All 384 samples were obtained over the course of a 3-day outreach clinic and CBC's were analyzed within 24 h of collection. Although there is limited outbreeding in the Amish community, no consanguineous marriages were identified. Sample processing, aliquoting, and freezing when indicated were performed immediately on site at each clinic. CBC analyses were performed within 24 h for all individuals and consisted of RBC, HB, HCT, MCV, MCH, MCHC, RDW, PLT, and WBC. RBC, HB, and MCV were directly measured, whereas HCT, MCH, and MCHC were mathematically derived from directly measured erythrocyte traits (MCV × RBC, HB/RBC, and MCH/MCV, respectively) (Walters and Abelson 1996). RDW is a measure of the variation of erythrocyte volume as evaluated by MCV and is defined by an inverse relationship between the standard deviation of MCV and the mean MCV. Informed consent was obtained following institutional guidelines and according to the Declaration of Helsinki.

### Genome-wide scan

DNA was extracted with the Gentra Puregene Kit (Qiagen) and genotyping of pedigree members was completed by the NHLBI Mammalian Genotyping Service at Marshfield, WI (http://research.marshfieldclinic.org/genetics/home/index.asp), using Screening Set 16 with 400 short tandem repeat polymorphic (STRP) markers at a 10 centiMorgan (cM) average interval. This generated a total of approximately 160,000 genotypes for the 384 samples. Data were processed and linkage analysis data files were created. Mendelian inconsistencies were identified using the computer program PEDCHECK and resolved (O'Connell and Weeks 1998).

### *HFE* rs1800562 and *ABO* rs687289 genotyping

rs1800562 (*HFE* C282Y) and rs687289 (*ABO*) were genotyped in the Amish cohort using a commercially available 5′ allelic discrimination high throughput fluorescent genotyping assay (TaqMan, Life Technologies) (Ranade et al. 2001). Hardy–Weinberg equilibrium was assessed by observed allele frequency as well as comparing the number of individuals by expected and observed genotypes. Assessment of an association of rs180562 and rs687280 with CBC traits was conducted using Sequential Oligogenic Linkage Analysis Routines (SOLAR).

### Covariate, heritability, and linkage analyses

Variance components analysis was conducted in SOLAR (version 4.2.7) and the significance of age, gender, rs687289 (a proxy for the type O *ABO* allele), and *VWF* C4120T genotype as covariates for RBC, HB, HCT, MCV, MCH, MCHC, RDW, PLT, and WBC was assessed (Almasy and Blangero 1998). Heritability of the quantitative phenotypes was estimated using SOLAR assuming a polygenic model incorporating covariates found to be significantly associated with the analyzed trait. All individuals in the pedigree were included in the analysis. Multipoint variance component quantitative trait linkage analysis was also conducted in SOLAR. Because of the large size of this pedigree, the multipoint estimates of pairwise identity by descent (IBD) status that are required for SOLAR computation were obtained using the software package Loki (version 2.4.5.) (Heath 1997).

## Results

### Description of CBC traits and heritability estimates

Standard CBC analyses were conducted for 384 Amish pedigree members (Table 1). As gender is known to be associated with several erythrocyte traits, we described all CBC traits by gender (Garner et al. 2000). Males had a higher mean RBC, HB, HCT, and MCHC and lower mean WBC than females, which is consistent with previously reported findings. The Amish cohort consists of 74 individuals heterozygous for a C4120T mutation that causes VWD; hence, we also described CBC traits by homozygous (CC4120, wild type) versus heterozygous (C4120T, VWD) genotype (Table 1). There were no significant VWD mutation-specific mean differences for any CBC trait. Additionally, previous studies reported that RBC, HB, and HCT are highly correlated traits (Evans et al. 1999). Within the Amish cohort, we demonstrated strong correlations (≥0.80) between RBC, HB, and HCT as well as MCV and MCH (Table S1). We also identified weaker correlations (0.49 to 0.56) of HB and HCT with MCV and MCH as well as inverse correlations between RDW and HB, HCT, MCV, and MCH (Table S1).

**Table 1:** Comparison of CBC traits by gender and *VWF* mutation

	Age	RBC (×10^6^/mL)	HB (g/dL)	HCT (%)	MCV (fL)	MCH (pg)	MCHC (g/dL)	RDW (%)	PLT (x10^6^/μL)	WBC (x10^6^/μL)
Mean	21.2	4.8	13.8	40.4	84.3	28.8	34.1	13.1	301.4	7.1
Male (*N* = 188)	18.8	5.0	14.2	41.3^1^	83.5	28.7	34.3^1^	13.1	299.8	6.9^1^
Female (*N* = 196)	23.6	4.7	13.5	39.5	85.0	29.0	34.0	13.1	302.8	7.3
*VWF* C4120T (*N* = 71)	25.4	4.8	13.9	40.6	84.5	28.9	34.1	13.3	311.7	7.5
*VWF* C4120C (*N* = 313)	20.2	4.8	13.8	40.4	84.2	28.8	34.1	13.0	298.8	7.1
Standard Deviation	17.3	0.4	1.4	4.1	4.6	1.7	0.9	0.8	78.6	2.3
Male	16.6	0.4	1.6	4.5	4.3	1.5	0.8	0.7	82.7	2.3
Female	17.6	0.3	1.1	3.4	4.9	1.9	0.9	0.8	74.5	2.4
*VWF* C4120T	19.9	0.4	1.5	4.7	5.2	1.9	0.9	0.8	77.6	2.4
*VWF* CC41210	16.4	0.4	1.4	3.9	4.5	1.7	0.9	0.8	78.7	2.3

CBC, complete blood count; RBC, red blood count; HB, Hemoglobin; HCT, Hematocrit; MCV, mean corpuscular volume; MCH, mean corpuscular hemoglobin; MCHC, mean corpuscular hemoglobin concentration; RDW, red blood cell distribution width; PLT, platelet count; WBC, white blood cell count.

1 *P*-value <0.05 comparing male and female groups.

In the variance components analysis, age was significantly associated with all CBC traits (Table S2). Gender was significantly associated with RBC, HB, HCT, MCHC, and WBC. *VWF* 4120 genotype was not associated with any CBC trait and was excluded as a covariate from all subsequent analyses. For RBC, age squared was also significant. Significant heritability estimates were obtained for RBC, MCV, MCH, MCHC, RDW, PLT, and WBC, with MCHC, RDW, PLT, and WBC demonstrating the highest genetic variance components (0.48, 0.35, 0.57, and 0.41, respectively; Table S2). Within the Amish pedigree, the genetic variance components for HB or HCT were not significant. It is not entirely clear why heritability was low for these two traits. We verified this finding by using MENDEL. One could argue that this low heritability for HB or HCT could be related to the decreased genetic variation observed in genetic isolates such as the Amish or by the fact that since this family has VWD perhaps those values are affected by bleeding. However, that was not the case for other RBC traits or platelets. Therefore at this point we cannot explain the low heritability observed for these two traits.

### Genome-wide QTL linkage analysis of CBC traits

**Figure 1:**
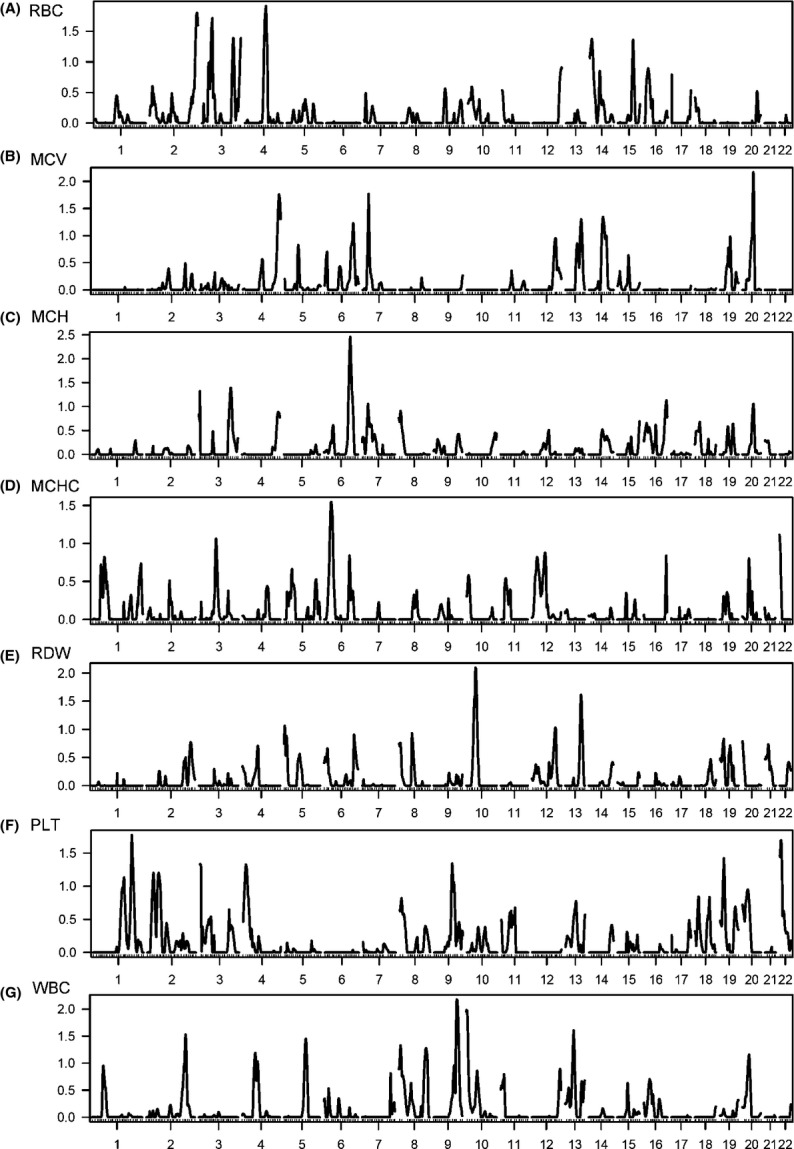
Genome-wide SOLAR QTL linkage scans. Quantitative trait locus linkage analysis was performed using SOLAR. The graphs represent the genome-wide scan results for (A) RBC, (B) MCV, (C) MCH, (D) MCHC, (E) RDW, (F) PLT, and (G) WBC and include age and gender as covariates when appropriate as indicated in Table 2. Somatic chromosomes are represented along the *x*-axis with breaks between each one, and chromosome numbers are listed in the center of the respective graph. LOD scores are represented along the *y*-axis with the scale of the *y*-axis changing relative to the maximum LOD score of the scan. As QTL analysis is a multipoint chromosome-wide analysis, the traces represent the LOD score as a continuous trace across the chromosome evaluated at each centiMorgan (cM). The maximum LOD score for single-trait analyses corresponds to MCH and maps to chromosome 6q25 (LOD = 2.46) followed by WBC on 9q33 (LOD = 2.18), MCV on 20q13 (LOD = 2.17), and RDW on 10p12 (LOD = 2.09).

**Figure 2:**
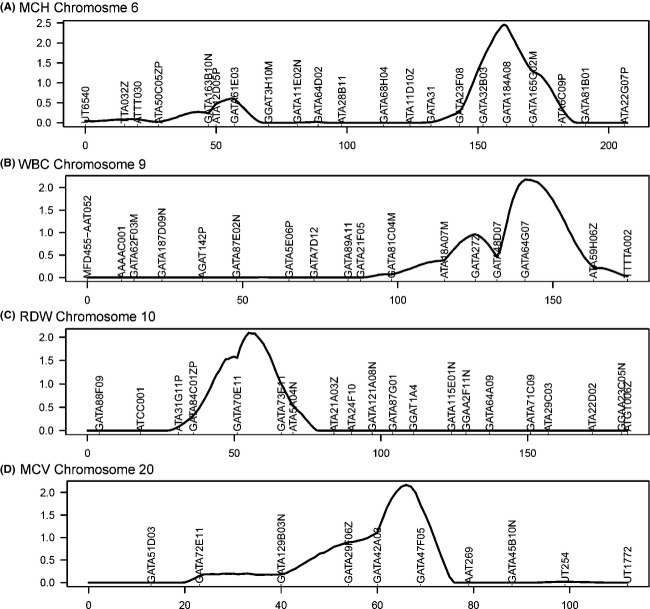
Chromosome-specific SOLAR QTL linkage scans for LOD scores greater than 2.0. Each panel represents a chromosome-wide scan for phenotypes where a candidate QTL was identified with a LOD score greater than 2.0. The map position along the chromosome is represented in cM along the *X*-axis and the LOD score is represented along the *Y*-axis. STRP marker names genotyped along each respective chromosome are imposed on the graphs vertically to represent the approximate coverage of each signal. Linkage traces are plotted continuously across a chromosome with an estimated LOD score at each cM. (A) Chromosome 6, MCH (LOD = 2.46). (B) Chromosome 9, WBC (LOD = 2.18). (C) Chromosome 10, RDW (LOD = 2.09). (D) Chromosome 20, MCV (LOD = 2.17).

Genome-wide QTL linkage analysis was conducted using SOLAR for RBC, MCV, MCH, MCHC, RDW, PLT, and WBC (Figure 1). Age was included as a covariate for all CBC traits and gender was included as a covariate for RBC, MCHC, and WBC (Table S2). HB and HCT were excluded, as there was no evidence for a significant genetic effect based on the heritability analysis. Four candidate loci with Logarithm of the odds (LOD) scores above 2.0 were identified (Table 2; Figure 2): 6q25 (MCH), 9q33 (WBC), 10p12 (RDW), and 20q13 (MCV). We also identified eleven candidate loci on eight chromosomes with LOD scores between 1.5 and <2.0 (Table 2): RBC (2q37.3, 3p22, 4q28), MCV (4q35, 7p15), MCHC (6p22), RDW (13q32), WBC (2q35, 10p15, 13q14), and PLT (22q11).

**Table 2:** Genome-wide QTL linkage analysis results, LOD score >1.5

Phenotype	Chromosome	1-LOD Interval (Mb)	Nearest Marker	LOD	*P*-value	% GCV^1^
PLT	1q32	208.28–227.02	GATA48B01	1.77	*P* = 0.003	
WBC	2q35	222.70–235.96	GATA4G12	1.53	*P* = 0.005	
RBC	2q37.3	242.01–243.20	GATA12H10, GATA178G09	1.80	*P* = 0.001	
RBC	3p22	26.72–41.17	GATA27C08	1.71	*P* = 0.001	
RBC	4q28	112.12–136.85	GATA62A12	1.91	*P* = 0.001	31.41
MCV	4q35	190.78–191.15	165zf8ZP	1.76	*P* = 0.001	
MCH	6q25	151.41–161.61	GATA184A08	2.46	*P* < 0.001	24.15
MCHC	6p22	16.00–38.78	GATA163B10	1.55	*P* = 0.003	
MCV	7p15	18.03–26.97	GATA41G07	1.77	*P* = 0.001	
WBC	9q33	121.94–133.23	GATA64G07	2.18	*P* = 0.002	13.40
WBC	10p15	0.00–4.93	GATA88F09	1.98	*P* = 0.002	
RDW	10p12	18.41–38.59	GATA70E11	2.09	*P* = 0.001	26.67
RDW	13q32	102.10–109.72	ATA26D07	1.61	*P* = 0.005	
WBC	13q14	47.06–77.57	GATA11C08	1.61	*P* = 0.005	
MCV-MCH	20p12.1–p11.23	11.14–48.64	GATA129B03	3.14	NA^2^	
MCV	20q13	44.00–48.51	GATA47F05	2.17	*P* < 0.001	37.41
PLT	22q11	0.00–23.22	GATA198B05	1.69	*P* = 0.004	

QTL, quantitative trait locus; PLT, platelet count; WBC, white blood cell count; RBC, red blood count; MCV, mean corpuscular volume; MCH, mean corpuscular hemoglobin; MCHC, mean corpuscular hemoglobin concentration; RDW, red blood cell distribution width.

1 Percent of the genetic variance component accounted for by the nearest marker. Calculated for linkage intervals with LOD scores greater than 2.0 and for the chromosome 4q28 RBC QTL.

2 SOLAR *P*-value simulation does not support bivariate analyses.

The 10p12 QTL for RDW represents one of the strongest linkage signals identified herein. Linkage between 9q33 and WBC also represents a novel finding. The candidate QTL for RBC that maps to 4q28 is a novel finding, although linkage of RBC to 4q31.2-q34.1 was reported in an Australian adolescent twin cohort (Table S3) (Iliadou et al. 2007). Additionally, we identified a candidate QTL with a LOD score suggestive of linkage for MCV, which maps to 4q35 (Table 2, Figure S1). These three linkage intervals for RBC and for MCV, which is inversely proportional to RBC, suggest the presence of a QTL on the telomeric half of the long arm of chromosome 4. The candidate linkage intervals for MCHC and MCH on chromosome 6 (6p22 and 6p25, respectively) and MCV on chromosome 20 (20q13) are consistent with previously reported QTL (Table S3). All other suggestive linkage intervals in Table 2 represent novel evidence of candidate QTL.

Two candidate loci on chromosome 6 corresponding to 6p22 for MCHC and 6q25 for MCH were identified (Table 2). Ten associations with five erythrocyte measures were previously reported on chromosome 6p22, eight of which are to rs1800562, a nonsynonymous variant in the hemochromatosis gene, *HFE* (Table S3). rs1800562 has been associated with HB, HCT, and MCV in two meta-analyses of Caucasian cohorts (Ganesh et al. 2009; Lo et al. 2011). Additionally, rs1800562 associations have been reported with serum iron as well as transferrin traits (Benyamin et al. 2009). Fifty linkage and association signals of erythrocyte measures, including RBC, HCT, HB, MCH, MCHC, and MCV, have been reported to multiple loci on the long arm of chromosome 6 in multiple Caucasian cohorts (Iliadou et al. 2007; Yang et al. 2007; Ganesh et al. 2009; Lo et al. 2011). The Framingham Heart Study reported linkage and association of RBC to 6q25.3-q27 and 6q25.1, respectively, which map within the linkage interval for MCH reported here (Table S3). Additionally, for MCV, a LOD score weakly suggestive of linkage (LOD = 1.23) was identified on 6q25 overlapping with the MCH QTL (LOD = 2.46). Due to the strong correlation between MCV and MCH, we conducted bivariate QTL linkage analysis for MCV-MCH on chromosome 6. Bivariate analysis lowered the maximum LOD score achieved with MCH, suggesting 6q25 primarily impacts MCH.

**Figure 3:**
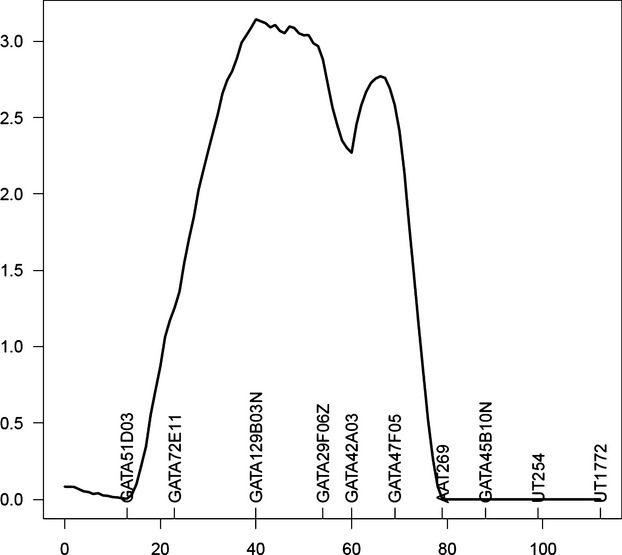
Chromosome 20 SOLAR QTL linkage scan for MCV-MCH bivariate analysis. The map position along the chromosome is represented in cM along the *X*-axis and the LOD score is represented along the *Y*-axis. STRP marker names genotyped along each respective chromosome are imposed on the graphs vertically to represent the approximate coverage of each signal. Linkage traces are plotted continuously across a chromosome with an estimated LOD score at each cM. The maximum LOD score of 3.14 maps to 20p12.1-p11.23 whereas the peak LOD score for the univariate analysis of MCV maps to the long arm of chromosome 20 (figure 2). Markers GATA29F06 and GATA42A03 flank the centromere, which corresponds to the trough within the 1-LOD candidate QTL interval.

Multiple associations between erythrocyte traits and loci on chromosome 20 have been reported (Yang et al. 2007; Ganesh et al. 2009). QTL analysis also identified weak evidence of linkage between 20q13 and MCH (LOD = 1.06). We therefore conducted bivariate analysis for MCV-MCH on chromosome 20, which resulted in a higher maximum LOD score of 3.14 (Table 2, Figure 3). When considered alone, the peak linkage region for MCV maps to 20q13 and the 1-LOD interval is restricted to the long arm of chromosome 20. Bivariate analysis of MCH-MCV shifted the peak linkage region to 20p12.1-p11.23. The 1-LOD interval of the bivariate QTL encompasses the centromere and the entire MCV QTL reported herein (Figure 2 and 3). RBC is inversely proportional to both MCV and MCH, and HB is proportional to MCH. Within Caucasian populations, an association between RBC and rs6108011 corresponds to the peak of the 20p12.1-p11.23 MCV-MCH linkage interval and an association between HB and rs6013509 (3 Mb telomeric to the 1-LOD linkage interval for MCV-MCH) (Yang et al. 2007; Ganesh et al. 2009).

### Proportion of variation attributable to candidate variants

To evaluate the proportion of variation accounted for by the candidate QTL reported here, we employed an internal function of SOLAR that estimates the proportion of variance associated with a linkage element (denoted as H2q1) for each marker. Table 2 lists the STRP marker nearest the peak LOD score that was used to estimate H2q1 for the corresponding QTL as well as the percent of the genetic variance component (GVC) accounted for by the marker listed. From this analysis, we determined that 6q25 (GATA184A08) accounted for 24.15% of the variation in MCH, 9q33 (GATA64G07) accounted for 13.40% of the variation in WBC, 10p12 (GATA70E11) accounted for 26.67% of the variation in RDW, and 20q13 (GATA47F05) accounted for 37.41% of the variation in MCV. Furthermore, on chromosome 4, 4q28 (GATA62A12Z) accounted for 24.15% of the variation in RBC and 4q35 (165zf8ZP) accounted for 31.41% of the variation in MCV.

### *HFE* C282Y is associated with MCH and MCHC

rs1800562, which has been associated erythrocyte traits in multiple studies (Table S3), corresponds to the amino acid change C282Y in *HFE*, the most common causal variant of hereditary hemochromatosis (MIM #235200). Within the Amish cohort, rs1800562 was found to be in Hardy–Weinberg equilibrium with a minor allele frequency of 0.16. With adjustment for age and gender, there was a significant association of rs1800562 genotype with MCH (*P* = 0.0036) and MCHC (*P* = 0.0056), but not with RBC (*P* = 0.4966), HB (*P* = 0.0666), HCT (*P* = 0.5241), MCV (*P* = 0.3354), or RDW (*P* = 0.1025).

## Discussion

CBC traits are multifactorial phenotypes determined by environmental and genetic components. Several genes have been implicated in red cell biology due to the identification of rare mutations in patients with Mendelian phenotypes. However, common variants in these same and other genes are likely involved in modulating more mild variation in hematologic phenotypes. In this study in a large Amish pedigree, which is expected to be environmentally more homogeneous, we found novel QTL that will allow for the discovery of genetic variants involved in erythropoiesis and red cell physiology.

The largest linkage peak corresponded to MCV-MCH on chromosome 20 and mapped to 20p12.1-20p11.23. This region contains several candidate genes. *SEC23B* is a COPII vesicle coat component involved in ER-to-Golgi trafficking. Mutations in *SEC23B* have been identified in several familial cases of congenital dyserythropoietic anemia (CDA) type II, a form of anemia due to ineffective erythropoiesis that is characterized by phenotypic variability, although interestingly, these patients do not have evident abnormalities in the MCV or MCH (Schwarz et al. 2009). A morpholino knockdown of *sec23b* in zebrafish resulted in aberrant erythropoiesis (Schwarz et al. 2009). Additionally in this region is *BMP2*, which maps to 20p12.3 (telomeric of the 1-LOD linkage interval reported herein), and encodes a bone morphogenetic protein that is part of the transforming growth factor-beta superfamily. Although not yet implicated in erythropoiesis, a significant association was demonstrated between rs235756 in *BMP2* and ferritin levels in a study of genetic modifiers of hemochromatosis. (Milet et al. 2007). Interestingly, *E2F1* maps to 20q11.22, also within the 1-LOD interval reported herein, and encodes a component of cell cycle regulation that is mediated by BMP2 (Tomari et al. 2005) It remains unclear whether *BMP2*, *E2F1*, or both may be QTL that affect iron-dependent traits such as MCV and MCH. *XKR7*, a Kell blood group complex subunit; *EPB41L1*, a neuronal variant of *EPB41*; and *HCK* (hemopoietic cell kinase), a member of the Src tyrosine kinase family primarily expressed in myeloid and B-lymphoid cell lineages, are also contained within the 1-LOD interval but are less likely modulators of erythrocyte traits. *EPB41*, which maps to chromosome 1p35.3, encodes an erythrocyte membrane protein (erythrocyte membrane protein band 4.1) that complexes with actin and spectrin provide shape to the discoid erythrocyte (Fairbanks et al. 1971; Correas et al. 1986).

Previous studies reported evidence for the *ABO* gene on chromosome 9q34 as a candidate QTL for several erythrocyte traits (Lin et al. 2005; Lo et al. 2011). We identified linkage of WBC to chromosome 9q33 (Table 2), but we did not identify concomitant linkage for erythrocyte traits. In characterizing the Amish pedigree for VWD, ABO blood types were determined by sequencing (see supporting information). Type O is overrepresented within the Amish cohort and type B is underrepresented. Approximately 60% of pedigree members were found to have type A blood (8% AA, 52% AO) and 31% of pedigree members were found to have type O blood. All other blood types, including type B and type AB, were rare among the Amish pedigree members (0.3–4%). Thus, analyses of blood types with CBC traits were limited to homozygous type A (AA), heterozygous type A (AO), and homozygous type O (OO). No significant differences in CBC trait means were identified between blood group genotypes (data not shown). Additionally, rs687289, a surrogate genetic marker for the type O *ABO* allele, was previously genotyped within the Amish cohort. Within SOLAR, rs687289 was evaluated as a covariate of CBC traits, and no association was found with WBC (*P* = 0.258) or other erythrocyte traits (Table S2).

Another region of interest is the short arm of chromosome 6 that contains several gene clusters, including transfer RNA genes, histone cluster component genes, and the major histocompatibility complex gene cluster. Two candidate genes within the linkage interval for MCHC include *HFE*, to which multiple MCH, HB, and HCT associations have been reported (Table S3); and *FANCE*, a Fanconi anemia gene. *HFE* encodes an MCH class-I type gene that regulates serum iron levels by increasing the dissociation constant of transferrin from the transferrin receptor complex, which reduces transferrin-mediated iron uptake within the gastrointestinal tract, as well as inhibiting iron efflux from monocyte and macrophage cells (Feder et al. 1998; Drakesmith et al. 2002) rs1800562, which has been associated with multiple erythrocyte traits in multiple populations, corresponds to a cysteine to tyrosine mutation at position 282 (C282Y), the most common mutation underlying the autosomal recessive disease hereditary hemochromatosis. Hemochromatosis is characterized by systemic iron overload but also anemia of chronic disease due to iron withholding from maturing erythrocytes (Feder et al. 1996). C282Y is a common variant with an estimated prevalence of 5–10% among populations of northern European ancestry and rare among other ethnic groups (Ajioka et al. 1997). In the Amish pedigree, we identified a higher MAF of approximately 16%, which may be due to sampling bias or may reflect a higher prevalence of the C282Y variant among Amish isolates. Healthy cohorts screened for C282Y status and evaluated for evidence of hemochromatosis or iron overload-related disease demonstrate a range in the prevalence of clinically significant hemochromatosis from less than 1% to 28%, estimates which have been disputed as too low and too high, respectively (Andersen et al. 2004; Allen et al. 2008). Thus, it is reasonable to hypothesize that C282Y, as well as other common variants within *HFE*, may also contribute to complex iron-dependent traits, such as the erythrocyte measures MCH and MCHC.

Other peaks with potential candidate genes within the interval were found in chromosome 4. On 4q28 three genes involved in cellular replication and differentiation, though not directly implicated in erythropoiesis, demonstrate relatively high expression in hematopoietic cell lineages, including early erythroid cells (GNF Expression Atlas 2 Data from U133A and GNF1H Chips, UCSC Genome Browser; http://www.genome.uscs.edu): *MAD2L1*, *EXOSC9*, and *CCNA2*. *MAD2L1* encodes a component of the mitotic spindle assembly checkpoint and is homologous to *MAD2*, a checkpoint component that stabilizes kinetochore-microtubule attachments (Kabeche and Compton 2012). Cyclin A2 (*CCNA2*) is a mammalian cell cycle regulator that promotes G1/S and G2/M transitions, and siRNA-mediated knockdown of *CCNA2* in K562 cells resulted in a significant decrease in erythroid differentiation following doxorubicin administration (Wang et al. 2009). In low doses, doxorubicin induces erythroid differentiation of K562 cells (human leukemic cell lineage), suggesting *CCNA2* may play a role as a key regulator of certain cell differentiation pathways, including erythropoiesis.

Additionally, we found a QTL with a LOD score suggestive of linkage for MCV that maps to 4q35 (Table 2) and contains *MLF1IP* which encodes MDS/myeloid leukemia factor 1 (MLF1) interacting protein, a component of the kinetochore complex. Overexpression of *Hls7*, the mouse ortholog of *MLF1*, results in suppression of erythropoietin-induced differentiation of the J2E erythroleukemic cell line, suggesting *MLF1* and its binding partners (including *MLF1IP*) may play a role in suppression of erythrocyte maturation (Williams et al. 1999). While the causal gene on chromosome 4 remains unclear, the two linkage intervals for RBC and MCV reported herein and the previous report of linkage of RBC strongly suggest the presence of a QTL for RBC on the telomeric half of the long arm of chromosome 4. Interestingly, we obtained a LOD score of 2.09 for RDW on 10p.12 that explains 26.67% of the variance. No evident candidate genes were identified in this interval and among all studies recently published on genetic determinants of CBC traits, none has reported a significant linkage signal for this particular trait (See Table S4).

In summary, we characterized a large Amish pedigree and identified several chromosomal regions of linkage to CBC traits. We also demonstrated association between rs1800562 and MCH and MCHC, confirming *HFE* and its most common causal variant of hereditary hemochromatosis as a candidate QTL of erythrocyte traits. These newly discovered regions that are likely to harbor QTL genes will provide insight into the genetic component underlying hematopoiesis and could have important implications for understanding the genetic basis of related disorders. Furthermore, a more complete elucidation of the variation of CBC traits will allow a more accurate characterization of the association of CBC traits with common diseases.

Although it is clear that the field of quantitative genetics has evolved into genome-wide association studies (GWAS) and whole-genome sequencing, linkage studies in large pedigrees like the one presented here will likely allow investigators to focus the search for rare variants amidst the noise encountered in the large amounts of data generated by these new technologies. A recent report by Desch et al. underscores this concept. In two young healthy large cohorts, making use of some of the sibling structure within the cohorts, they found a strong linkage signal in chromosome 2 for von Willebrand Factor levels that had not been detected by association in the same group, suggesting that aggregates of rare variants may explain some of the heritability for that trait (Desch et al. 2013). Therefore, linkage studies will still be useful in guiding large sequencing analysis for the detection of rare variants that may explain part of the heritability of complex traits like the ones described here.

## Supporting Information

**Data S1.** ABO blood group.

**Figure S1.** Chromosome 4 SOLAR QTL linkage scans RBC and MCV univariate analyses.

**Table S1.** Correlation of CBC traits.

**Table S2.** Significance of covariates and heritability estimates for CBC traits.

**Table S3.** Previously published replication of candidate QTL identified in the Amish Pedigree.

**Table S4.** Literature review of candidate QTL of CBC traits.
