# Supplementary material for: Quantitative trait locus linkage analysis in a large Amish pedigree identifies novel candidate loci for erythrocyte traits
Source: Mol Genet Genomic Med. 2013 May 31;1(3):131–41. doi: 10.1002/mgg3.16 (PMC3775389; doi:10.1002/mgg3.16)
Supplement: Supplementary file 1 [file mgg30001-0131-SD1.docx]

**Supporting Information**

**ABO blood group**

Sequencing of exons 6 and 7 of the ABO gene was performed in all collected samples. Genotype calls were made based on haplotypes formed by sequence variants at positions 261, 802, 803 and 1069.[^1^](#_ENREF_1) Although this is not standard for ABO determination, the advantages of DNA sequencing over serum antibody detection include the possibility of detecting multiple alleles and the ability to distinguish individuals heterozygous for an O allele (AO and BO) from individuals with no O allele (AA and BB) that are indistinguishable by serologic classification alone. The genotyping of ABO was validated by sequencing 10 samples from the University of Iowa Blood Bank with known serologic ABO status.

**References**

1. Pearson SL, Hessner MJ. A(1,2)BO(1,2) genotyping by multiplexed allele-specific PCR. *Br J Haematol*. 1998;100:229-234

**Supplemental Figure 1. Chromosome 4 SOLAR QTL linkage scans RBC and MCV univariate analyses.** The map position along the chromosome is represented in cM along the X-axis and the LOD score is represented along the Y-axis. STRP marker names genotyped along each respective chromosome are imposed on the graphs vertically to represent the approximate coverage of each signal. Linkage traces are plotted continuously across a chromosome with an estimated LOD score at each cM.

**Supporting Table 1. Correlation of CBC traits**

| Phenotype | RBC | HB | HCT | MCV | MCH | MCHC | RDW | PLT | WBC |
| --- | --- | --- | --- | --- | --- | --- | --- | --- | --- |
| RBC | 1 | 0.80 | 0.82 | 0.04 | 0.05 | 0.06 | -0.15 | -0.18 | -0.19 |
| HB | 0.80 | 1 | 0.97 | 0.54 | 0.56 | 0.24 | -0.42 | -0.37 | -0.30 |
| HCT | 0.82 | 0.97 | 1 | 0.56 | 0.49 | 0.05 | -0.41 | -0.32 | -0.22 |
| MCV | 0.04 | 0.54 | 0.56 | 1 | 0.89 | -0.03 | -0.55 | -0.32 | -0.19 |
| MCH | 0.05 | 0.56 | 0.49 | 0.89 | 1 | 0.34 | -0.11 | -0.23 | -0.19 |
| MCHC | 0.06 | 0.24 | 0.05 | -0.03 | 0.34 | 1 | -0.11 | -0.23 | -0.19 |
| RDW | -0.15 | -0.42 | -0.41 | -0.55 | -0.52 | -0.11 | 1 | 0.19 | 0.10 |
| PLT | -0.18 | -0.37 | -0.32 | -0.32 | -0.37 | -0.23 | 0.19 | 1 | 0.51 |
| WBC | -0.19 | -0.30 | -0.22 | -0.19 | -0.26 | -0.19 | 0.10 | 0.51 | 1 |

Within the Amish cohort, we demonstrated strong correlations (R^2^≥0.80) between RBC, HB, and HCT as well as MCV and MCH. We also identified moderate correlations (R^2^ range: 0.49 to 0.56) of HB and HCT with MCV and MCH as well as moderate inverse correlations between RDW and HB, HCT, MCV, and MCH (R^2^ range: -0.41 to -0.55).

**Supporting Table 2. Significance of covariates and heritability estimates for CBC traits**

|  | Age  p-value | Gender  p-value | C4120T^*^  p-value | rs687289^†^  p-value | Heritability | Heritability  p-value |
| --- | --- | --- | --- | --- | --- | --- |
| RBC | 7.005x10^-14^ | 2.478x10^-21^ | 0.119 | 0.258 | 0.23 | 0.00675 |
| HB | 6.056x10^-39^ | 2.699x10^-19^ | 0.312 | 0.267 | 0.12 | 0.0792 |
| HCT | 2.435x10^-33^ | 2.750x10^-16^ | 0.229 | 0.596 | 0.04 | 0.215 |
| MCV | 5.482x10^-31^ | 0.153 | 0.553 | 0.716 | 0.20 | 0.00213 |
| MCH | 6.135x10^-25^ | 0.841 | 0.789 | 0.840 | 0.22 | 0.00916 |
| MCHC | 0.01881 | 1.056x10^-4^ | 0.813 | 0.663 | 0.48 | 1.060x10^-9^ |
| RDW | 1.773x10^-13^ | 0.411 | 0.134 | 0.462 | 0.35 | 5.103x10^-9^ |
| PLT | 1.682x10^-13^ | 0.0972 | 0.994 | 0.348 | 0.57 | 3.140x10^-12^ |
| WBC | 1.238x10^-7^ | 7.601x10^-3^ | 0.427 | 0.258 | 0.41 | 2.360x10^-11^ |

^*^ *VWF* C4120T mutation

^†^ rs687289 SNP in *ABO*, a surrogate marker of ABO blood type

| Phenotype | SNP | Chromosome | Gene | Statistics | Study |
| --- | --- | --- | --- | --- | --- |
| RBC |  | 4q31.2-q34.1 |  | LOD=3.07 | Iliadou *et al*.([Iliadou*, et al* 2007](#_ENREF_23)) |
| HB | rs1800562 | 6p22.1 | *HFE* | p=5.74x10^-19^ | Ganesh *et al*.([Ganesh*, et al* 2009](#_ENREF_18)) |
| HB | rs1800562 | 6p22.1 | *HFE* | p=1.40x10^-15^ | Lo *et al*.([Lo*, et al* 2011](#_ENREF_27)) |
| HCT | rs1800562 | 6p22.1 | *HFE* | p=2.04x10^-9^ | Ganesh *et al*.([Ganesh*, et al* 2009](#_ENREF_18)) |
| HCT | rs1800562 | 6p22.1 | *HFE* | p=2.50x10-10 | Lo *et al*.([Lo*, et al* 2011](#_ENREF_27)) |
| MCH | rs1800562 | 6p22.1 | *HFE* | p=2.76x10^-9^ | Kullo *et al.*([Kullo*, et al* 2010](#_ENREF_25)) |
| MCV | rs1800562 | 6p22.1 | *HFE* | p=1.40x10^-23^ | Soranzo *et al.*([Soranzo*, et al* 2009](#_ENREF_38)) |

**Supporting Table 3. Previously published replication of candidate QTL identified in the Amish Pedigree**

**Supporting Table 4. Literature review of candidate QTL of CBC traits**

| **Chromosome** | **Trait** | **Population** | **Statistics** | **Notes** | **PMID** |
| --- | --- | --- | --- | --- | --- |
| **1p34.3** | **RBC** | **Caucasian** | **4.20E-06** | ***FLJ11730; BC016328*** | **17903294** |
| **1q21** | **Hct** | **Mice** | **LOD 3.9** | **Locus named *Hctq1*, recesive model** | **16596451** |
| **1q22** | **MCHC** | **Japanese** | **3.39E-09** | ***BGLAP-PAQR6-SMG5-TMEM79-C1orf85-VHLL-CCT3-C1orf182*** | **20139978** |
| **1q23.2** | **HB** | **Caucasian** | **1.60E-07** | ***OR10J1; OR10J5*** | **17903294** |
| **1q23.1** | **MCHC** | **Caucasian** | **p=1.033E-10** | ***SPTA1*** | **19862010** |
| **1q23.2** | **WBC** | **African American** | **LOD 96.8** | ***DARC*** | **18179887** |
| **1q23.2** | **WBC** | **African American** | **1.40E-103** | ***DARC*** | **21153663** |
| **1q24.3** | **MPV** | **Caucasian** | **2.10E-14** | ***DNM3*** | **19820697** |
| **1q31.3** | **MCH** | **Japanese** | **6.76E-10** | ***ATP6V1G3-PTPRC*** | **20139978** |
| **1q32.1** | **MPV** | **Caucasian** | **1.40E-20** | ***TMCC2*** | **19820697** |
| **1q44** | **HB** | **Caucasian** | **LOD 2.04** |  | **16950815** |
| **1q44** | **HCT** | **Caucasian** | **LOD 2.72** |  | **16950815** |
| **1q44** | **MCV** | **Japanese** | **2.33E-08** | ***TRIM58*** | **20139978** |
| **2p11.2-q11.2** | **MCV** | **Caucasian** | **LOD1.63** |  | **16950815** |
| **2p16.2** | **HB** | **Caucasian** | **1.00E-06** |  | **17903294** |
| **2p16.1** | **MCV** | **Caucasian** | **p=1.125E-14** | ***BCL11A*** | **19862010** |
| **2p21** | **HB** | **Caucasian** | **p=7.052E-13** | ***PRKCE*** | **19862010** |
| **2p21** | **HCT** | **Caucasian** | **p=3.748E-15** | ***PRKCE*** | **19862010** |
| **2p21** | **MPV** | **Caucasian** | **3.20E-11** | ***EHD3*** | **19820697** |
| **2p21** | **RBC** | **Japanese** | **3.81E-08** | ***PRKCE*** | **20139978** |
| **3p14.2-p13** | **RBC** | **Caucasian** | **LOD 2.2** |  | **16950815** |
| **3p21–p13** | **MPV** | **Caucasian** | **5.50E-31** | ***ARHGEF3*** | **19820697** |
| **3p23-p22.3** | **RDW-PLT** | **Baboons** | **LOD 1.6** |  | **17557178** |
| **3p24.2** | **MCH** | **Japanese** | **3.52E-10** | ***THRB*** | **20139978** |
| **3p24.2** | **MCV** | **Japanese** | **3.33E-08** | ***THRB*** | **20139978** |
| **3q27.1** | **PLT** | **Japanese** | **5.38E-11** | ***THPO-CHRD*** | **20139978** |
| **3q29** | **MCH** | **Japanese** | **4.46E-11** | ***TFRC-ZDHHC19*** | **20139978** |
| **3q29** | **MCH** | **Caucasian** | **p=7.729E-13** | ***TFRC*** | **19862010** |
| **3q29** | **MCV** | **Japanese** | **1.66E-08** | ***TFRC-ZDHHC19*** | **20139978** |
| **3q29** | **MCV** | **Caucasian** | **p=8.499E-14** | ***TFRC*** | **19862010** |
| **3q29** | **MCV** | **Caucasian** | **6.40E-04** | ***TFRC-ZDHHC19*** | **21153663** |
| **4p12-q13.3** | **RBC** | **Caucasian** | **LOD 2.5** |  | **17903294** |
| **4p15.1-p14** | **MCV** | **Caucasian** | **LOD 1.73** |  | **16950815** |
| **4p15.33** | **RBC** | **Caucasian** | **4.50E-06** |  | **17903294** |
| **4q12** | **MCH** | **Japanese** | **2.80E-25** | ***PDGFRA-HK1*** | **20139978** |
| **4q12** | **MCV** | **Japanese** | **2.19E-29** | ***PDGFRA-HK1*** | **20139978** |
| **4q12** | **MCV** | **Caucasian** | **p=9.816E-10** | ***KIT*** | **19862010** |
| **4q12** | **RBC** | **Japanese** | **1.92E-17** | ***PDGFRA-HK1*** | **20139978** |
| **4q31.2-q34.1** | **RBC** | **Caucasian** | **LOD 3.07** |  | **16950815** |
| **5p15.33** | **RBC** | **Japanese** | **3.01E-08** | ***TERT*** | **20139978** |
| **5q21.1** | **MCH** | **Caucasian** | **5.80E-06** |  | **17903294** |
| **6p21.1** | **MCH** | **Japanese** | **1.80E-20** | ***USP49-MED20-BYSL-CCND3*** | **20139978** |
| **6p21.1** | **MCH** | **Caucasian** | **p=8.198E-20** | ***CCND3/BYSL*** | **19862010** |
| **6p21** | **MCV** | **Australian, Dutch** | **1.20E-09** | ***CCND3*** | **19853236** |
| **6p21.1** | **MCV** | **Japanese** | **3.62E-27** | ***USP49-MED20-BYSL-CCND3*** | **20139978** |
| **6p21.1** | **MCV** | **Caucasian** | **p=1.121E-31** | ***CCND3/BYSL*** | **19862010** |
| **6p21.1** | **MCV** | **Caucasian** | **7.00E-19** | ***BYSL/ CCND3*** | **19820697** |
| **6p21.1** | **RBC** | **Japanese** | **1.09E-10** | ***USP49-MED20-BYSL-CCND3*** | **20139978** |
| **6p21** | **WBC** | **Caucasian** | **1.90E-08** |  | **21153663** |
| **6p21.3** | **MCV** | **Caucasian** | **1.40E-23** | ***HFE*** | **19820697** |
| **6p21.31** | **PLT** | **Japanese** | **6.66E-11** | ***BAK1*** | **20139978** |
| **6p21.3** | **PLT** | **Caucasian** | **3.70E-10** | ***BAK1*** | **19820697** |
| **6p21.31** | **PLT** | **African American** | **6.20E-08** | ***BAK1*** | **21153663** |
| **6p21.31** | **PLT** | **Caucasian** | **7.60E-73** | ***BAK1*** | **21153663** |
| **6p21.33** | **WBC** | **Japanese** | **6.76E-09** | ***CDSN-PSORS1C1*** | **20139978** |
| **6p22** | **HB** | **Caucasian** | **p=5.737E-19** | ***HFE*** | **19862010** |
| **6p22.1** | **HB** | **Caucasian** | **1.40E-15** | ***HFE*** | **21153663** |
| **6p22** | **HCT** | **Caucasian** | **p=2.035E-9** | ***HFE*** | **19862010** |
| **6p22.1** | **HCT** | **Caucasian** | **2.50E-10** | ***HFE*** | **21153663** |
| **6p22.2** | **MCH** | **Caucasian** | **p=3.868E-39** | ***SLC17A3*** | **19862010** |
| **6p22.1** | **MCH** | **Emergency department** | **2.76E-09** | ***HFE*** | **20927387** |
| **6p22.2** | **MCH** | **Emergency department** | **4.66E-08** | ***SLC17A1*** | **20927387** |
| **6p22.2** | **MCHC** | **Japanese** | **5.00E-10** | ***SLC12A7*** | **20139978** |
| **6p22** | **MCV** | **Caucasian** | **p=1.012E-46** | ***HFE*** | **19862010** |
| **6p22.1** | **MCV** | **Caucasian** | **6.80E-16** | ***HFE*** | **21153663** |
| **6q21** | **MCH** | **Japanese** | **1.49E-08** | ***C6orf182-CD164*** | **20139978** |
| **6q21** | **MCV** | **Japanese** | **2.51E-08** | ***C6orf182-CD164*** | **20139978** |
| **6q21** | **MCV** | **Caucasian** | **p=4.198** | **CD164** | **19862010** |
| **6q21** | **RBC** | **Japanese** | **6.92E-09** | ***C6orf182-CD164*** | **20139978** |
| **6q21** | **RBC** | **Caucasian** | **LOD 1.73** |  | **16950815** |
| **6q23.1-q23.2** | **HB** | **Caucasian** | **LOD 3.03** |  | **16950815** |
| **6q23.3** | **HCT** | **Caucasian** | **p=2.811E-15** | ***HBS1L-MYB*** | **19862010** |
| **6q23-24** | **HCT** | **Caucasian** | **LOD 3.4** | ***EBP41L2, HEBP2 HPFH*** | **15635079** |
| **6q23.3** | **HCT** | **Caucasian** | **5.50E-04** | ***MYB*** | **21153663** |
| **6q23.1-q23.2** | **HCT** | **Caucasian** | **LOD 2.95** |  | **16950815** |
| **6q23.3** | **Ht** | **Japanese** | **9.52E-11** | ***HBS1L-MYB*** | **20139978** |
| **6q23.3** | **MCH** | **Japanese** | **2.82E-66** | ***HBS1L-MYB*** | **20139978** |
| **6q23.3** | **MCH** | **Caucasian** | **p=7.356E-69** | ***HBS1L-MYB*** | **19862010** |
| **6q23.3** | **MCH** | **Emergency department** | **5.17E-16** | ***HBS1L/MYB*** | **20927387** |
| **6q23.3** | **MCH** | **Emergency department** | **3.12E-14** | ***HBS1L/MYB*** | **20927387** |
| **6q23.3** | **MCH** | **Emergency department** | **4.94E-14** | ***HBS1L/MYB*** | **20927387** |
| **6q23.3** | **MCH** | **Emergency department** | **1.36E-15** | ***HBS1L/MYB*** | **20927387** |
| **6q23.3** | **MCH** | **Emergency department** | **7.05E-11** | ***HBS1L/MYB*** | **20927387** |
| **6q23.3** | **MCH** | **Emergency department** | **1.42E-08** | ***HBS1L/MYB*** | **20927387** |
| **6q23.3** | **MCHC** | **Japanese** | **6.22E-12** | ***HBS1L-MYB*** | **20139978** |
| **6q23.3** | **MCHC** | **Caucasian** | **p=6.486E-10** | ***HBS1L-MYB*** | **19862010** |
| **6q23.3** | **MCV** | **Japanese** | **3.44E-56** | ***HBS1L-MYB*** | **20139978** |
| **6q23.3** | **MCV** | **Caucasian** | **p=7.241E-86** | ***HBS1L-MYB*** | **19862010** |
| **6q23.3** | **MCV** | **Emergency department** | **1.37E-14** | ***HBS1L/MYB*** | **20927387** |
| **6q23.3** | **MCV** | **Emergency department** | **5.03E-13** | ***HBS1L/MYB*** | **20927387** |
| **6q23.3** | **MCV** | **Emergency department** | **7.94E-13** | ***HBS1L/MYB*** | **20927387** |
| **6q23.3** | **MCV** | **Emergency department** | **2.82E-15** | ***HBS1L/MYB*** | **20927387** |
| **6q23.3** | **MCV** | **Emergency department** | **2.50E-10** | ***HBS1L/MYB*** | **20927387** |
| **6q23.3** | **MCV** | **Emergency department** | **3.49E-08** | ***HBS1L/MYB*** | **20927387** |
| **6q23 –q24** | **MCV** | **Caucasian** | **7.40E-42** | ***HBS1L -MYB*** | **19820697** |
| **6q23.3** | **MCV** | **Caucasian** | **3.90E-09** | ***MYB*** | **21153663** |
| **6q23.3** | **PLT** | **Japanese** | **2.54E-14** | ***HBS1L-MYB*** | **20139978** |
| **6q23.3** | **RBC** | **Japanese** | **7.31E-48** | ***HBS1L-MYB*** | **20139978** |
| **6q23.3** | **RBC** | **Caucasian** | **p=1.148E-47** | ***HBS1L-MYB*** | **19862010** |
| **6q23.3** | **RBC** | **Emergency department** | **1.11E-14** | ***HBS1L/MYB*** | **20927387** |
| **6q23.3** | **RBC** | **Emergency department** | **2.46E-13** | ***HBS1L/MYB*** | **20927387** |
| **6q23.3** | **RBC** | **Emergency department** | **2.85E-13** | ***HBS1L/MYB*** | **20927387** |
| **6q23.3** | **RBC** | **Emergency department** | **7.18E-12** | ***HBS1L/MYB*** | **20927387** |
| **6q23.3** | **RBC** | **Emergency department** | **5.57E-09** | ***HBS1L/MYB*** | **20927387** |
| **6q23.3** | **RBC** | **Caucasian** | **4.70E-04** | ***MYB*** | **21153663** |
| **6q23.1-q23.2** | **RBC** | **Caucasian** | **LOD 1.88** |  | **16950815** |
| **6q23.3** | **WBC** | **Japanese** | **1.67E-09** | ***HBS1L-MYB*** | **20139978** |
| **6q23.3** | **WBC** | **Caucasian** | **4.40E-04** | ***MYB*** | **21153663** |
| **6q24.1** | **MCH** | **Japanese** | **1.42E-09** | ***CITED2*** | **20139978** |
| **6q24.1** | **MCH** | **Caucasian** | **p=1.262E-17** | ***CITED2*** | **19862010** |
| **6q24.1** | **MCV** | **Australian, Dutch** | **5.30E-09** |  | **19853236** |
| **6q24.1** | **MCV** | **Japanese** | **1.08E-09** | ***CITED2*** | **20139978** |
| **6q24.1** | **MCV** | **Caucasian** | **p=4.665E-25** | ***CITED2*** | **19862010** |
| **6q25.1** | **RBC** | **Caucasian** | **6.30E-06** | ***MAP3K7IP2*** | **17903294** |
| **6q25.3-q27** | **RBC** | **Caucasian** | **LOD 2.9** |  | **17903294** |
| **7p12.2** | **MCV** | **Caucasian** | **p=4.689E-13** | ***IKZF1*** | **19862010** |
| **7q11.21-q11.22** | **HB** | **Caucasian** | **LOD 1.55** |  | **16950815** |
| **7q21.2** | **WBC** | **Japanese** | **2.44E-08** | ***CDK6*** | **20139978** |
| **7q21.2** | **WBC** | **Caucasian** | **4.10E-04** | ***CDK6*** | **21153663** |
| **7q22.1** | **HCT** | **Caucasian** | **p=4.45E-10** | ***TFR2*** | **19862010** |
| **7q22** | **HCT** | **Caucasian** | **4.30E-07** | ***TFR2*** | **21153663** |
| **7q22.1** | **MCV** | **Caucasian** | **p=2.543E-11** | ***TFR2*** | **19862010** |
| **7q22** | **MCV** | **Caucasian** | **1.60E-07** | ***TFR2*** | **21153663** |
| **7q22.3** | **MPV** | **Caucasian** | **1.60E-33** | ***PIK3CG*** | **19820697** |
| **7q22.1** | **RBC** | **Caucasian** | **p=1.123E-9** | ***EPO*** | **19862010** |
| **7q22** | **RBC** | **Caucasian** | **4.90E-10** | ***TFR2*** | **19820697** |
| **7q22** | **RBC** | **Caucasian** | **1.60E-05** | ***TFR2*** | **21153663** |
| **7q22.1** | **RBC** | **Caucasian** | **1.30E-06** | ***EPO*** | **21153663** |
| **7q36.1** | **HB** | **Caucasian** | **p=3.025E-15** | ***PRKAG2*** | **19862010** |
| **7q36.1** | **HB** | **Caucasian** | **1.60E-10** | ***PRKAG2*** | **21153663** |
| **7q36.1** | **HCT** | **Caucasian** | **p=6.045E-15** | ***PRKAG2*** | **19862010** |
| **7q36.1** | **HCT** | **Caucasian** | **5.00E-09** | ***PRKAG2*** | **21153663** |
| **7q36.1-q36.2** | **MCV** | **Caucasian** | **LOD 2.2** |  | **17903294** |
| **7q36.2** | **MCV** | **Caucasian** | **LOD 1.71** |  | **16950815** |
| **7q36.2-q36.3** | **MCV** | **Caucasian** | **LOD 2.14** |  | **16950815** |
| **8p12** | **RBC** | **Caucasian** | **LOD 1.52** |  | **16950815** |
| **8p21.3** | **MCV** | **Japanese** | **3.32E-08** | ***DOK2-XPO7*** | **20139978** |
| **8q22.3-q24.11** | **RBC** | **Caucasian** | **LOD 1.99** |  | **16950815** |
| **8q24.13** | **HB** | **Caucasian** | **2.10E-06** |  | **17903294** |
| **8q24.13-q24.11** | **HCT** | **Caucasian** | **LOD 2.02** |  | **16950815** |
| **9p24.1** | **MCH** | **Japanese** | **5.69E-11** | ***RCL1*** | **20139978** |
| **9p24.1** | **MCH** | **Caucasian** | **p=2.166E-14** | ***RCL1*** | **19862010** |
| **9p24.1** | **MCV** | **Japanese** | **2.50E-14** | ***RCL1*** | **20139978** |
| **9p24.1** | **MCV** | **Caucasian** | **p=3.184E-20** | ***RCL1*** | **19862010** |
| **9p24.2** | **MCV** | **Caucasian** | **LOD 1.61** |  | **16950815** |
| **9p24.1** | **PLT** | **Japanese** | **2.95E-13** | ***RCL1*** | **20139978** |
| **9p24.1 –p24.3** | **PLT** | **Caucasian** | **8.50E-17** | ***AK3*** | **19820697** |
| **9p24.2** | **RBC** | **Caucasian** | **LOD 1.62** |  | **16950815** |
| **9q34.2** | **Hb** | **Japanese** | **1.18E-11** | ***ABO*** | **20139978** |
| **9q34.2** | **HB** | **Caucasian** | **9.60E-04** | ***ABO*** | **21153663** |
| **9q34** | **HCT-HB** | **Caucasian** | **LOD 3.1** |  | **15635079** |
| **9q34.2** | **Ht** | **Japanese** | **6.06E-10** | ***ABO*** | **20139978** |
| **9q34.2** | **MCHC** | **Japanese** | **4.32E-08** | ***ABO*** | **20139978** |
| **9q34** | **MCV** | **Korean** | **5.06E-05** | ***ABO*. Identified a CNV where deletion associated with MCV.** | **22963146** |
| **9q34.2** | **RBC** | **Japanese** | **3.33E-12** | ***ABO*** | **20139978** |
| **9q34** | **RBC** | **Korean** | **2.13E-06** | ***ABO*** | **22963146** |
| **10p11.21** | **MCH** | **Caucasian** | **5.90E-06** | ***ANKRD30A*** | **17903294** |
| **10q11.21** | **MCH** | **Japanese** | **3.82E-12** | ***ALOX5-MARCH8-ANUBL1-FAM21C-AGAP4*** | **20139978** |
| **10q11.23** | **MCH** | **Japanese** | **6.42E-10** | ***MSMB-NCOA4-TIMM23*** | **20139978** |
| **10q11.21** | **MCV** | **Japanese** | **2.60E-11** | ***ALOX5-MARCH8-ANUBL1-FAM21C-AGAP4*** | **20139978** |
| **10q11.23** | **MCV** | **Japanese** | **6.73E-09** | ***MSMB-NCOA4-TIMM23*** | **20139978** |
| **10q11.21** | **MCV** | **Caucasian** | **p=1.346E-10** | ***Gene: MARCH8*** | **19862010** |
| **10q11.21** | **MCV** | **Caucasian** | **4.10E-04** | ***ALOX5-MARCH8-ANUBL1-FAM21C-AGAP4*** | **21153663** |
| **10q22.1** | **HB** | **Caucasian** | **p=2.116E-11** | ***HK1*** | **19862010** |
| **10q22.1** | **HCT** | **Caucasian** | **p=9.636E-14** | ***HK1*** | **19862010** |
| **10q21.2 –q21.3** | **MPV** | **Caucasian** | **3.30E-21** | ***JMJD1C*** | **19820697** |
| **11p15.4** | **MCH** | **Caucasian** | **LOD 3.6** | **Hemoglobin B gene cluster** | **17211848** |
| **11p15.3-p15.1** | **MCV** | **Caucasian** | **LOD 3.3** |  | **17903294** |
| **11p15.4** | **MCV** | **Caucasian** | **LOD 3.8** | **Hemoglobin B gene cluster** | **17211848** |
| **11p15.4** | **MCV** | **African American** | **2.90E-04** | ***HBB*** | **21153663** |
| **11p15.4** | **MCV** | **African American** | **4.90E-07** | ***MMP26*** | **21153663** |
| **11p15.5** | **MPV** | **Caucasian** | **1.30E-14** | ***BET1L*** | **19820697** |
| **11p15.3-p15.2** | **RBC** | **Caucasian** | **LOD 3.2** |  | **17903294** |
| **11q12.1** | **MCH** | **Caucasian** | **6.90E-08** | ***OR5AP2; OR5AR1; OR9G1; OR9G4*** | **17903294** |
| **12p13.32** | **RBC** | **Japanese** | **6.44E-09** | ***CCND2*** | **20139978** |
| **12p13.2** | **RBC** | **Caucasian** | **LOD 3.0** |  | **17211848** |
| **12p13.32-p13.31** | **RBC** | **Caucasian** | **LOD 2.8** |  | **17903294** |
| **12q13.13** | **PLT** | **African American** | **1.60E-06** | ***NFE2-COPZI*** | **21153663** |
| **12q13.13** | **RBC** | **Caucasian** | **LOD 1.63** |  | **16950815** |
| **12q15** | **WBC** | **Japanese** | **2.85E-08** | ***RAP1B-NUP107-SLC35E3*** | **20139978** |
| **12q23.1** | **RBC** | **Caucasian** | **LOD 1.61** |  | **16950815** |
| **12q24.13** | **HB** | **Caucasian** | **p=1.159E-11** | ***TRAFD1*** | **19862010** |
| **12q24.12** | **HB** | **Caucasian** | **3.20E-07** | ***SH2B3*** | **21153663** |
| **12q24.12** | **HCT** | **Caucasian** | **p=1.363E-12** | ***SH2B3-ATXN2*** | **19862010** |
| **12q24.12** | **HCT** | **Caucasian** | **1.50E-06** | ***SH2B3*** | **21153663** |
| **12q24.14** | **MCHC** | **Japanese** | **7.25E-10** | ***ALDH2*** | **20139978** |
| **12q24.31** | **MPV** | **Caucasian** | **2.70E-44** | ***WDR66*** | **19820697** |
| **12q24.12** | **PLT** | **Japanese** | **4.75E-19** | ***SH2B3*** | **20139978** |
| **12q24** | **PLT** | **Caucasian** | **2.20E-13** | ***ATXN2*** | **19820697** |
| **12q24** | **PLT** | **Caucasian** | **7.70E-12** | ***PTPN11*** | **19820697** |
| **13q22.1** | **MCV** | **Caucasian** | **LOD 1.74** |  | **16950815** |
| **14q23.3** | **MCV** | **Caucasian** | **p=4.907E-8** | ***FNTB*** | **19862010** |
| **14q24.3-q32.12** | **RBC** | **Caucasian** | **LOD 2.6** |  | **17903294** |
| **15q22.31** | **MCH** | **Japanese** | **2.88E-09** | ***IGDCC4-DPP8-PTPLAD1-C15orf44-SLC24A1-DENND4A*** | **20139978** |
| **15q22.1** | **MPV** | **Caucasian** | **1.90E-08** | ***TPM1*** | **19820697** |
| **15q26.3** | **RBC** | **Caucasian** | **LOD 2.3** |  | **17903294** |
| **16p13.3** | **HB** | **African American** | **6.10E-08** | ***HBA2-HBA1*** | **21153663** |
| **16p13.3** | **HB** | **African American** | **1.30E-06** | ***MMP25*** | **21153663** |
| **16p13.3** | **MCH** | **Japanese** | **2.83E-09** | ***HBA2-HBA1-LUC7L-ITFG3-RGS11*** | **20139978** |
| **16p13.3** | **MCH** | **Caucasian** | **p=2.675E-10** | ***ITFG3*** | **19862010** |
| **16p13.3** | **MCHC** | **African American** | **7.40E-13** | ***HBA2-HBA1*** | **21153663** |
| **16p13.3** | **MCV** | **Caucasian** | **p=1.819E-12** | ***ITFG3*** | **19862010** |
| **16p13** | **MCV** | **African American** | **5.00E-07** |  | **21153663** |
| **16p13.3** | **MCV** | **African American** | **1.40E-33** | ***HBA2-HBA1*** | **21153663** |
| **16q24.3** | **MCHC** | **Japanese** | **3.55E-13** | ***CDT1*** | **20139978** |
| **17p13.1-p12** | **MCV** | **Caucasian** | **LOD 1.95** |  | **16950815** |
| **17p13.2** | **PLT** | **Japanese** | **2.13E-12** | ***GP1BA*** | **20139978** |
| **17q11.2-q22** | **HB** | **Caucasian** | **LOD 2.61** |  | **16950815** |
| **17q11.2-q22** | **HCT** | **Caucasian** | **LOD 2.98** |  | **16950815** |
| **17q11.2** | **MPV** | **Caucasian** | **1.40E-22** | ***TAOK1*** | **19820697** |
| **17q12** | **WBC** | **Caucasian** | **9.40E-09** | ***GSDMA/ ORMDL3*** | **19820697** |
| **17q21.32-q22** | **RBC** | **Caucasian** | **LOD 2.05** |  | **16950815** |
| **17q21.1** | **WBC** | **Japanese** | **2.94E-14** | ***GSDMA-PSMD3-CSF3-MED24*** | **20139978** |
| **17q21.1** | **WBC** | **Caucasian** | **1.80E-19** | ***CSF3*** | **21153663** |
| **17q25.1-q25.3** | **RBC** | **Caucasian** | **LOD 2.3** |  | **17903294** |
| **18p11.32-p11.31** | **RBC** | **Caucasian** | **LOD 3.3** |  | **17903294** |
| **18q22.3** | **MPV** | **Caucasian** | **1.40E-10** | ***CD226*** | **19820697** |
| **19p13.2** | **MCH** | **Caucasian** | **p=1.415E-11** | ***GCDH*** | **19862010** |
| **19p13.2** | **MCV** | **Caucasian** | **p=2.173E-12** | ***RTBDN*** | **19862010** |
| **19q13.43** | **MCV** | **Caucasian** | **8.30E-07** | ***GALP*** | **21153663** |
| **19p13.12** | **PLT** | **African American** | **3.00E-07** | ***TPM4*** | **21153663** |
| **19p13.12** | **PLT** | **Caucasian** | **3.00E-07** | ***TPM4*** | **21153663** |
| **19p13.3** | **RBC** | **Caucasian** | **LOD 3.2** | ***EPOR, EKLF*** | **17211848** |
| **20p12.3** | **RBC** | **Caucasian** | **5.80E-06** |  | **17903294** |
| **20p13** | **MPV** | **Caucasian** | **7.70E-11** | ***SIRPA*** | **19820697** |
| **20q13.2** | **HB** | **Caucasian** | **p=1.054E-10** | ***TSHZ2*** | **19862010** |
| **20q13.31** | **MCV** | **Japanese** | **1.37E-08** | ***RBM38*** | **20139978** |
| **22q11.21** | **MCV** | **Japanese** | **1.04E-08** | ***HIC2-UBE2L3*** | **20139978** |
| **22q11.21-q11.22** | **MCV** | **Caucasian** | **LOD 1.7** |  | **16950815** |
| **22q11.23-q13.2** | **RBC** | **Caucasian** | **LOD 2.2** |  | **17903294** |
| **22q12 –q13** | **MCV** | **Caucasian** | **4.30E-10** | ***FBXO7*** | **19820697** |
| **22q12.1-q12.3** | **MCV** | **Caucasian** | **LOD 2.16** |  | **16950815** |
| **22q12.3** | **HB** | **Australian** | **5.30E-07** | ***TMPRSS6*, also associated with iron status (mechanism)** | **19820699** |
| **22q12.3** | **Hb** | **Japanese** | **1.64E-10** | ***TMPRSS6*** | **20139978** |
| **22q12.3** | **HB** | **Caucasian** | **p=3.25E-25** | ***TMPRSS6*** | **19862010** |
| **22q12.3** | **HB** | **Caucasian** | **1.60E-13** | ***TMPRSS6,* V736A substitution in serine protease domain reported w/ dose effect** | **19820698** |
| **22q12.3** | **HCT** | **Caucasian** | **p=1.846E-13** | ***TMPRSS6*** | **19862010** |
| **22q12.3** | **MCH** | **Japanese** | **4.84E-25** | ***TMPRSS6*** | **20139978** |
| **22q12.3** | **MCH** | **Caucasian** | **p=8.77E-34** | ***TMPRSS6*** | **19862010** |
| **22q12.3** | **MCH** | **Emergency department** | **1.10E-12** | ***TMPRSS6*** | **20927387** |
| **22q12.3** | **MCH** | **Emergency department** | **3.73E-08** | ***TMPRSS6*** | **20927387** |
| **22q12.3** | **MCH** | **Emergency department** | **2.41E-11** | ***TMPRSS6*** | **20927387** |
| **22q12.3** | **MCH** | **Caucasian** | **9.50E-10** | ***TMPRSS6*** | **19820697** |
| **22q12.3** | **MCHC** | **Japanese** | **7.73E-14** | ***TMPRSS6*** | **20139978** |
| **22q12.3** | **MCHC** | **Emergency department** | **2.40E-11** | ***TMPRSS6*** | **20927387** |
| **22q12.3** | **MCHC** | **Emergency department** | **1.13E-12** | ***TMPRSS6*** | **20927387** |
| **22q12.3** | **MCV** | **Australian** | **1.10E-10** | ***TMPRSS6*, also associated with iron status (mechanism)** | **19820699** |
| **22q12.3** | **MCV** | **Japanese** | **1.22E-15** | ***TMPRSS6*** | **20139978** |
| **22q12.3** | **MCV** | **Caucasian** | **p=2.772E-41** | ***TMPRSS6*** | **19862010** |
| **22q12.3** | **MCV** | **Emergency department** | **5.41E-09** | ***TMPRSS6*** | **20927387** |
| **22q13.33** | **MCH** | **Japanese** | **4.37E-08** | ***NCAPH2-SCO2-TYMP-KLHDC7B*** | **20139978** |
| **22q13.33** | **MCV** | **Caucasian** | **p=1.033E-15** | ***ECGF1*** | **19862010** |
| **22q13.33** | **MCV** | **Caucasian** | **1.00E-06** | ***ECGF1*** | **21153663** |
| **Xp21.3-p11.4** | **MCV** | **Caucasian** | **LOD 2.3** |  | **17903294** |
| **Xq25** | **HB** | **African American** | **1.20E-15** | ***G6PD*** | **21153663** |
| **Xq25** | **HCT** | **African American** | **1.20E-13** | ***G6PD*** | **21153663** |
| **Xq25** | **MCV** | **African American** | **3.00E-18** | ***G6PD*** | **21153663** |
| **Xq25** | **RBC** | **African American** | **4.30E-21** | ***G6PD*** | **21153663** |

**Supporting Figure 1**

**
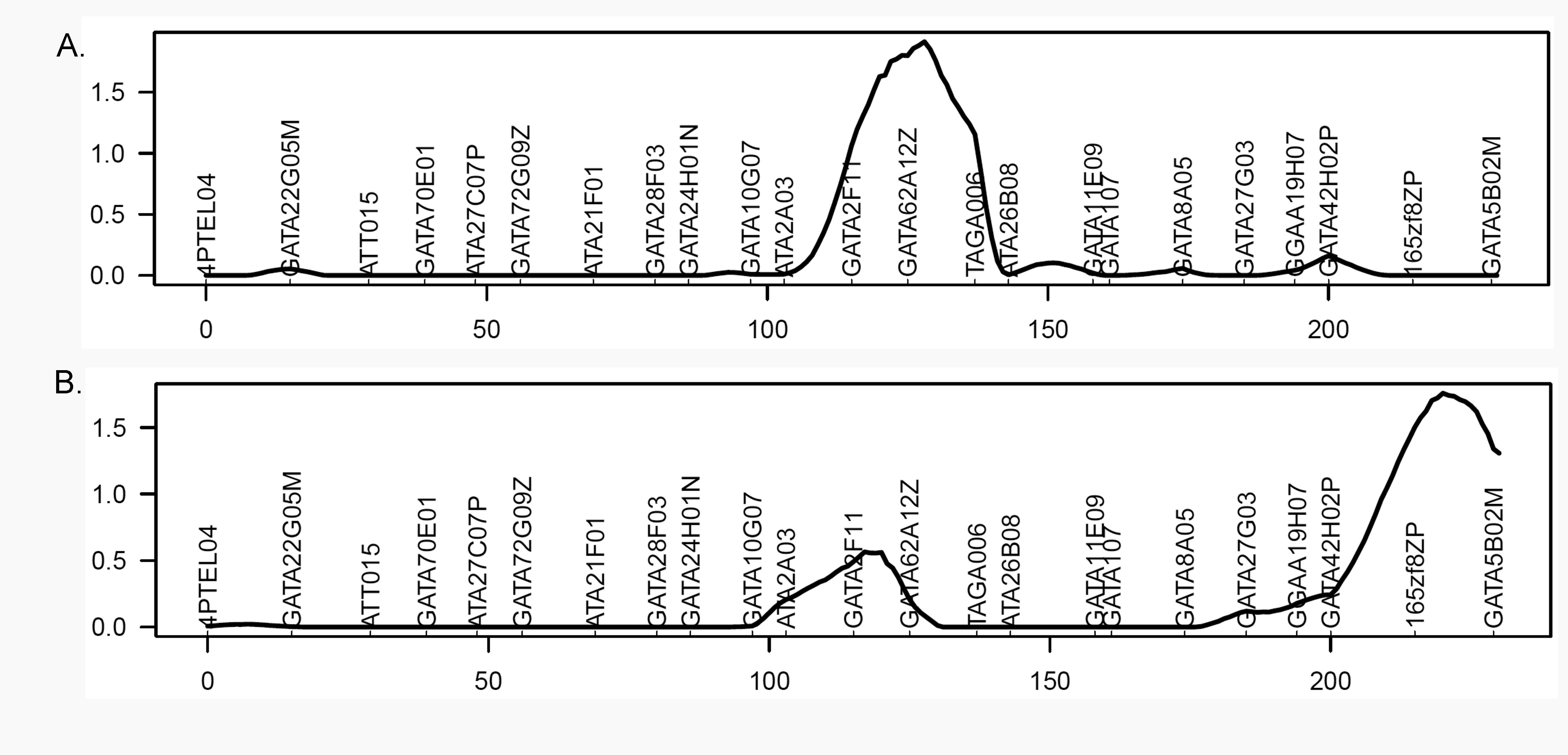
**
